# Inhibitor potency and assay conditions: A case study on SARS-CoV-2 main protease

**DOI:** 10.1073/pnas.2106095118

**Published:** 2021-09-02

**Authors:** Mira A. M. Behnam, Christian D. Klein

**Affiliations:** ^a^Medicinal Chemistry, Institute of Pharmacy and Molecular Biotechnology, Heidelberg University, 69120 Heidelberg, Germany

Li et al. (1) report known drugs as inhibitors of the main protease (M^pro^) of severe acute respiratory syndrome coronavirus 2 (SARS-CoV-2). The compounds, including atazanavir, were initially identified by virtual screening, followed by fluorescence resonance energy transfer (FRET)-based biochemical inhibition assays.

In this letter, we demonstrate that the inhibitory activity achieved in enzymatic assays by the compounds is sensitive to the conditions used. This observation supports the proposed conformational selection paradigm for SARS-CoV-2 M^pro^ (2).

Using an M^pro^ with C-terminal His-tag and the FRET substrate Abz-VVTLQ/SGDap(Dnp)R-OH (3), atazanavir showed no or minimal inhibition under all studied conditions, including the buffer used by Li et al. (1) This point was previously raised by Ma and Wang (4), and, in comparison to their substrate Dabcyl-KTSAVLQ/SGFRKME(Edans) (5), our shorter substrate renders an influence of substrate length less likely and pinpoints the difference in activity to the His-tagged M^pro^ construct.

Ma and Wang (4) and Ma et al. (6) suggest that 1,4-dithiothreitol (DTT) affects inhibitory activity, as it would maintain M^pro^ in a reduced state and eliminate nonspecific thiol-reactive compounds. We screened a number of buffers with varying composition and pH using boceprevir, a reported inhibitor for SARS-CoV-2 M^pro^ (6). Among these buffers are those reported by Li et al. (1) and Ma et al. (6) Notably, we found a fourfold difference in the potency of boceprevir, depending on the choice of assay buffer (Fig. 1*D*). Factors that influence activity include pH, ionic strength, and polyols. Boceprevir showed fourfold higher inhibition in the presence of 20% glycerol in comparison to a buffer with the same pH containing 150 mM NaCl. Mass spectrometry indicated formation of an adduct between M^pro^ and boceprevir. The *K*_m_ of the substrate varied by almost twofold for the previously mentioned conditions. In addition to the effect on the substrate and inhibitor activity, a change of the pH profile of the substrate at pH 8 was observed in the presence of 150 mM NaCl.

**Fig. 1.**
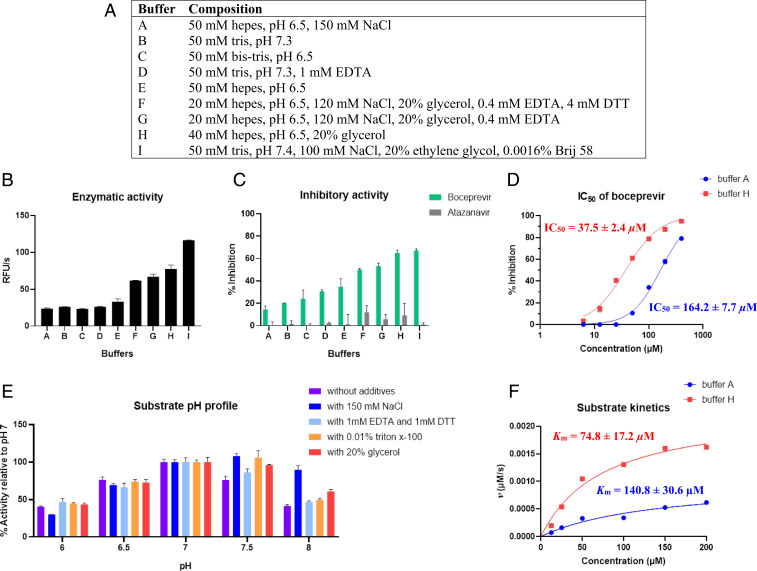
(*A*) List of buffers, EDTA is ethylenediaminetetraacetic acid. (*B*) Processing of the substrate Abz-VVTLQ/SGDap(Dnp)R-OH (20 µM) by SARS-CoV-2 M^pro^ (0.5 µM) in different buffers as relative fluorescence units per second (RFU/s). (*C*) Inhibitory activity of boceprevir and atazanavir (50 µM) in different buffers, [substrate] = 20 µM, [enzyme] = 0.5 µM. (*D*) Dose–response curves for M^pro^ inhibition by boceprevir in buffers A and H, [substrate] = 20 µM, [enzyme] = 0.5 µM, IC_50_ stands for half-maximal inhibitory concentration. (*E*) The pH profile of the substrate Abz-VVTLQ/SGDap(Dnp)R-OH in buffers with variable additives. Buffer concentration is 50 mM except for buffers with 20% glycerol, where the concentration is 40 mM. Bis-Tris buffer for pH 6 and 6.5; Tris buffer for pH 7, 7.5, and 8. (*F*) Michaelis−Menten curves for the substrate Abz-VVTLQ/SGDap(Dnp)R-OH in buffers A and H, *v* stands for reaction rate.

These findings can be partially explained by pH-dependent conformational changes, previously described for SARS-CoV and SARS-CoV-2 M^pro^. Furthermore, salinity was reported to influence the monomer−dimer equilibrium (2). The same factors were shown, by NMR studies, to affect conformational dynamics in other viral proteases, such as the protease from flaviviruses (7).

Li et al. (1) discuss the reliability and suitability of experimental methods used for in vitro assays in the identification of compounds with different binding mechanisms to the target (5). We would like to add that, even within the domain of biochemical assays, testing conditions can have a profound effect on inhibitory activity. The distinctive intramolecular interaction profiles of compounds with their targets naturally lead to a variable sensitivity toward screening conditions. Certain types of molecular recognition, such as electrostatic interactions, will be expected to have a higher sensitivity toward ionic strength of the buffer, pH, and so forth. Eventually, decisions on compound priorization for further development must be guided by cellular and phenotypic assays (8), which can also help to pinpoint the most suitable biochemical environment and experimental approach for the identification of promising hits.

## Change History

September 8, 2021: The licensing has been updated.
